# Preheated Composite for Prosthetic Cementation to Enamel and Dentin: A Scoping Review

**DOI:** 10.3390/dj14010069

**Published:** 2026-01-21

**Authors:** Anca Labunet, Andreea Kui, Alexandra Vigu, Andrada Voina-Tonea, Alexandru Burde, Sorina Sava

**Affiliations:** 1 Dental Materials Discipline, Department 4—Prosthodontics and Dental Materials, Faculty of Dental Medicine, “Iuliu Hatieganu” University of Medicine and Pharmacy, 400012 Cluj-Napoca, Romania or jiglau.anca@umfcluj.ro (A.L.); andrada.tonea@umfcluj.ro (A.V.-T.); savasorina@elearn.umfcluj.ro (S.S.); 2 Prosthetic Dentistry Discipline, Department 4—Prosthodontics and Dental Materials, Faculty of Dental Medicine, “Iuliu Hatieganu” University of Medicine and Pharmacy, 400012 Cluj-Napoca, Romania; andreeakui@gmail.com; 3 Dental Technology Discipline, Department 2—Faculty of Nursing and Health Sciences, “Iuliu Hatieganu” University of Medicine and Pharmacy, 400012 Cluj-Napoca, Romania; burde.alexandru@umfcluj.ro

**Keywords:** preheated composite, composite resins, cementation, indirect restoration, dental luting

## Abstract

**Background and Objectives:** Preheated composite resins have been proposed as an alternative to conventional luting agents due to their improved resistance, color stability, and adaptation. This review aims to critically evaluate the current literature on the use of preheated composites as luting agents exclusively on dentin and enamel, focusing on their mechanical behavior, optical properties, and biological effects, in order to determine whether they provide superior clinical outcomes compared with conventional resin cements. **Materials and Methods:** A comprehensive literature search from 2015 to 2025 was conducted in accordance with PRISMA-ScR guidelines. Eligible studies included in vitro investigations comparing the preheated composite with other luting agents performed on human, bovine, analog dentin or enamel substrates. Studies meeting these criteria were screened, evaluated, and synthesized. **Results:** Fifteen studies met the inclusion criteria: nine focused on the mechanical performance, and the remaining six studies examined additional properties such as color stability, pulpal temperature changes during preheating, film thickness characteristics, and the influence on marginal discrepancy. **Conclusions**: Preheated composite resins offer improved mechanical properties, marginal adaptation, and fracture resistance compared with conventional luting agents. However, their performance is highly technique-sensitive, and clinical outcomes depend on operator skill, restoration thickness, and material selection. Preheating generally does not compromise color stability, but it can elevate pulpal temperature, particularly when residual dentin is thin. Overall, preheated composites have potential clinical advantages, provided that careful handling and appropriate application are ensured.

## 1. Introduction

Adhesion is a primary focus of contemporary research, as new materials and new techniques arise to fulfill the need for higher esthetics. As has been previously discussed, important differences in adhesion ability come about due to the enamel and dentin’s structural organization. Bonding to enamel is highly reliable due to its composition of 94–96% inorganic substances, 1–4% water, and 4–5% organic material. Enamel exhibits strong intermolecular forces and high surface energy, which favor bonding. In contrast, adhesion to dentin is more challenging because it is a porous, hydrated tissue composed of hydroxyapatite embedded in a collagen matrix, which compromises bonding. Dentin has lower intermolecular forces and surface energy, with a composition of 50–70% inorganic matter, 20–30% organic matter, and 10–20% water [1,2].

When luting highly esthetic, thin indirect restorations, resin cements—available in light-, chemical-, or dual-curing forms—are preferred for their high hardness, low solubility in oral fluids, and strong micromechanical adhesion to enamel and dentin [3]. However, resin composites, with a higher amount of inorganic filling and superior strength, may provide better results, significantly higher survival rates, and improved fracture resistance in veneers or similarly thin restorations [4].

Preheating composite resins has been proposed as an alternative to low-viscosity luting agents. Heating composite resins to a temperature of around 54–68 °C reduces their viscosity, making them flow more easily and allowing them to adapt better to the tooth surface and the inner surface of the restoration [5]. The flowability approaches that of resin cement, ensuring complete seating of the restoration without gaps [6,7,8,9]. Various preheating techniques, devices, temperatures, heating durations, and transport methods have been described in the literature [10].

Preheated resin cementation provides superior marginal adaptation, reducing microleakage and enhancing restoration durability [11]. It also aids in cementing restorations with lower polymerization shrinkage and a higher conversion rate [12,13]. However, this outcome is technique-sensitive due to the need for precise temperature control, although this should not cause irreversible pulpal damage [14]. Preheated composites provide better wear resistance [15], fracture toughness, and longevity than conventional resin cements, and they are especially beneficial for indirect restorations in high-stress areas [4,16].

Another important advantage of using preheated composite materials is the simplification of inventory and ensuring consistent optical and mechanical behavior across direct and indirect restorations. Preheated composites come in a wide range of shades and translucencies, allowing for better color matching and esthetic integration.

There are, however, certain limitations to preheated composite use, as the working time is reduced by the cooling and thickening of the material as the temperature decreases [12]. Preheating devices are required, as well as the use of a compatible bonding protocol and thorough light curing, especially with thicker or opaque restorations [8]. To prevent thermal injury to the pulp, it is important to preserve dentin and avoid a continuous high-energy photo-curing technique [17]. Beyond the protective role of interposed materials and the remaining dentin thickness, factors such as pulpal blood circulation, tissue volume, and the perfusion of fluid within dentinal tubules and surrounding tissues also contribute significantly to heat dissipation and resistance against temperature increases [18]. A rise of 5.5 °C in pulpal temperature is considered capable of causing irreversible tissue change [19,20]. Although much of the heat generated during resin-based composite (RBC) polymerization is dissipated, the intrapulpal temperature may still surpass the threshold associated with pulpal damage [21]. Studies further emphasize that the remaining dentin thickness plays a pivotal role in moderating intrapulpal temperature increases due to its intrinsic heat-dissipating properties [22,23].

A scoping review published in 2023, focusing on physicochemical properties of preheated composite resins, concluded that most studies showed decreased viscosity, increased conversion degree and microhardness of composite resins, and better marginal adaptation of direct and indirect restorations. Also, flexural strength was not affected, and data regarding bond strength were inconclusive due to heterogeneity among studies [24]. In contrast to these results, a 2025 systematic review found that although preheated composites offer certain benefits, their mechanical properties and the thickness of the cementation layer do not surpass those of conventional resin cements [25]. These two recent studies included research performed on different bonded surfaces, such as plastic, composites, different ceramics and enamel. They did not focus on the influence of temperature variations on bonding or the substrate.

As a consequence of these opposing findings in recent research and their focus solely on mechanical behavior, this scoping review aims to compile and critically analyze the existing literature on the effects of preheating composite resins when applied exclusively to dentin and enamel surfaces, focusing on mechanical behavior, color stability, and pulpal temperature changes. Previous reviews did not address these specific issues and were conducted on varied surfaces, as opposed to our present research on dental surfaces only. A scoping review was chosen instead of a meta-analysis because the existing literature is heterogeneous in terms of study design, populations, interventions, and outcome measures, limiting the feasibility of meaningful quantitative synthesis. The aim of this review is to map the extent and nature of the evidence, clarify key concepts, and identify knowledge gaps rather than estimate pooled effect sizes. Consequently, a scoping review provides a more appropriate and methodologically sound approach.

The study is driven by the need to address the following research question: “Does the use of preheated composite as a luting agent on dentin and enamel provide superior outcomes in terms of mechanical, optical, and biological performance?” Accordingly, the null hypothesis is formulated as such: preheating the composite resin used as a luting agent on dentin and enamel does not result in any significant differences in mechanical performance, color stability, or pulpal temperature changes compared with conventional (non-preheated) composite techniques.

## 2. Materials and Methods

This scoping literature review was conducted in accordance with the PRISMA-ScR guidelines (PRISMA extension for scoping reviews). The research question was formulated using the PICOT framework (Population, Intervention, Comparison, Outcomes, and Time), as follows:

P: Preheated composite materials.
I: Cementation of prosthetic restorations in vitro on dental surfaces.
C: Comparison between preheated composite and other types of cements.
O: Mechanical properties, film thickness, color changes, and biological effects on dental tissues.
T: Studies published within the past 10 years.

### 2.1. Information Sources and Search Strategy

The literature search was carried out by two reviewers (AL and AV) between 2 October and 10 October 2025, using three bibliographic databases: Medline (PubMed), Scopus, and Embase. Four main search concepts were defined (Table 1), along with a comprehensive set of keywords and search terms—including MeSH terms—to ensure consistency across all databases. Detailed search strategies for each database are presented in Table 2. In addition to the database searches, which retrieved 101 articles, a manual search was conducted by reviewing the reference lists of relevant studies to identify any additional eligible articles—11 were identified.

### 2.2. Eligibility Criteria

Detailed inclusion and exclusion criteria are explained below in a list and in tabular form (Table 3).

Inclusion Criteria

1. Articles focusing on the comparison between preheated composite and other cement types.
2. In vitro studies performed on dentin or enamel substrate, either human, bovine or analog.
3. Studies published in English, completed between 2015 and 2025.

Exclusion Criteria

1. Studies that did not include at least two groups, with one group using a preheated composite, or that lacked a comparative analysis.
2. Clinical studies, reviews.
3. Studies evaluating bonding to composite or plastic surfaces, rather than to human or analog dentin or enamel.
4. Articles published in languages other than English or published more than 10 years ago.

### 2.3. Data Extraction and Method of Analysis

Data extraction was performed using a standardized form, with all information recorded in an Excel spreadsheet (v.15.17, Microsoft, Redmond, WA, USA). The extracted data included bibliographic details, study design and methodology, key findings, and conclusions.

To ensure consistency and minimize bias, two reviewers (AL and AV) independently carried out the data extraction. Any discrepancies were resolved through discussion or, when necessary, by consulting a third reviewer (AK). A standardized template was used to document essential study details, including experimental groups and results relevant to the review objectives. The initial data extraction was conducted by the primary reviewers and subsequently verified for accuracy and completeness by two additional investigators.

## 3. Results

### Data Collection

**Figure 1:**
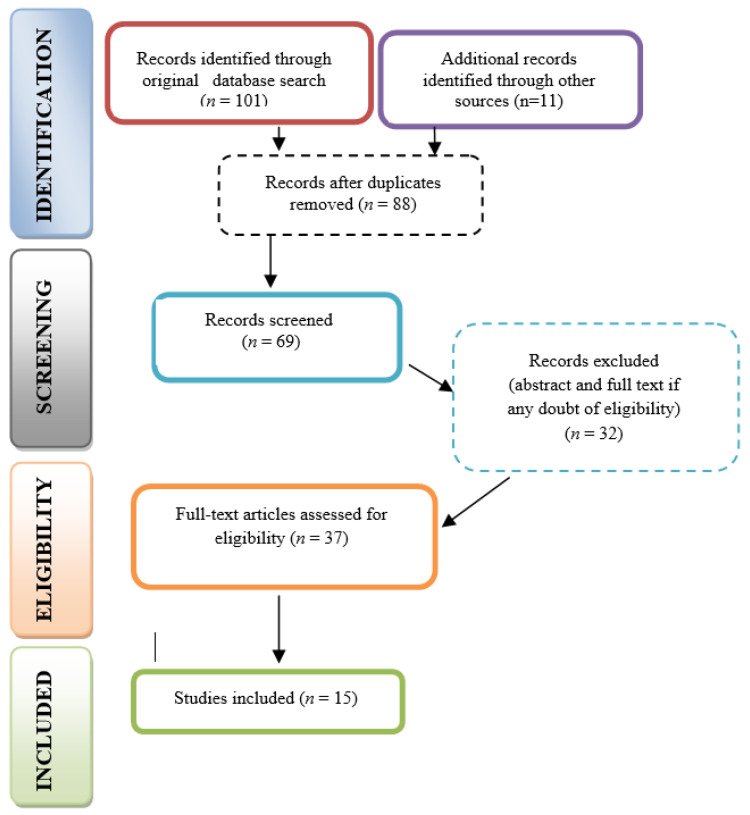
PRISMA flow diagram.

The initial search strategy, summarized in Table 1 and Table 2, identified 112 articles. After removing duplicates, 88 unique records remained. Applying the inclusion and exclusion criteria reduced this number to 69 articles eligible for screening. In the first phase, titles and abstracts were evaluated for relevance to the research question, resulting in 37 articles that proceeded to a full-text eligibility review. Any disagreements during this process were resolved through discussion or, when necessary, consultation with a third and fourth reviewer (AK, SS). After full text reading, articles were excluded according to exclusion criteria—mainly for a lack of comparison or no dental surfaces being included. Ultimately, 15 studies met the criteria and were included in the final review. The PRISMA flow diagram provides an overview of the study selection process and inclusion criteria (Figure 1), PRISMA checklist also provided in the Supplementary Files.

Nine studies explored the mechanical behavior of the preheated composite for luting different materials to dentine and/or enamel, as shown in Table 2. Six studies, presented in short form in Table 4, focused on other issues related to preheated composite use, such as color stability, pulpal temperature changes, film thickness, and marginal discrepancy.

Several studies reported higher fracture resistance and microtensile bond strength when preheated composites were used, especially in combination with immediate dentin sealing or increased composite temperature. Light-cured resin cements showed higher shear bond strength in some settings, while dual-cured self-adhesive cements generally exhibited lower or comparable bond strength. Preheating was also associated with improved adaptation and thinner luting interfaces due to reduced viscosity.

However, findings were not entirely consistent. Some studies reported no significant differences in failure load or microleakage among luting agents, and polymerization effectiveness of preheated composites decreased with increasing restoration thickness. Overall, outcomes were influenced by substrate type, restoration thickness, and curing strategy, highlighting substantial methodological heterogeneity across studies.

As shown in Table 5, several studies focused on color-related outcomes and biological and interfacial outcomes. Resin cements and composite resins differed in their influence on the final color and color stability of lithium disilicate and zirconia restorations. Light-cured and dual-cured resin cements showed comparable color stability, whereas microfilled composite resins exhibited clinically relevant color changes over time. Preheating generally did not affect the degree of conversion or long-term color stability, although warming resin cements up to 54 °C improved the color stability of ceramic restorations under aging conditions.

Preheated composites were associated with higher intrapulpal temperature increases, particularly in deeper preparations, though values were influenced primarily by remaining dentin thickness. Marginal adaptation varied by luting agent, with flowable composites showing the least marginal discrepancy and preheated high-viscosity composites demonstrating the greatest marginal increase, in some cases exceeding clinically acceptable limits. Additionally, preheated composites required greater operator skill to achieve clinically acceptable film thickness.

Overall, these findings suggest that while preheating luting materials may offer certain advantages, it can also introduce challenges related to marginal accuracy, film thickness, and pulpal temperature, underscoring the need for careful material selection and clinical technique.

## 4. Discussion

Although scoping reviews are not primarily designed to assess the methodological quality of included studies, this review conducted a structured critical appraisal to better contextualize the strength and reliability of the available evidence. Each included study was evaluated for clarity of objectives, appropriateness of experimental design, adequacy of sample preparation, transparency in preheating protocols, and robustness of outcome measurements. Particular emphasis was placed on whether the studies used standardized heating devices, clearly reported composite temperatures, and ensured consistent handling techniques—factors known to influence viscosity, adaptation, and polymerization behavior.

Methodological quality varied across the evidence base. Several studies demonstrated strong experimental control, including well-defined heating procedures, appropriate grouping (with direct comparison between preheated composites and alternative cements), and the use of validated mechanical or optical testing methods. These studies provided higher confidence in their reported outcomes. However, some studies exhibited limitations such as small sample sizes, incomplete reporting of substrate characteristics, or inadequate details regarding adhesive protocols. In certain cases, temperature monitoring during preheating or during pulpal heating measurements was insufficiently described, reducing the interpretability of biological outcome data.

Variability was also observed in the use of dentin and enamel substrates, with some investigations relying on analog or bovine sources without providing justification or validation of their equivalence to human tissue. This introduced a potential source of heterogeneity. Additionally, the consistency of outcome measures differed across studies, with some employing standardized ISO testing procedures while others used laboratory-specific methods, limiting comparability.

Overall, the critical appraisal revealed that while many studies offer valuable insights into the effects of preheated composite as a luting agent, differences in methodological rigor must be taken into account when interpreting the collective findings. These variations highlight the need for more standardized protocols and more comprehensive reporting in future research.

### 4.1. Mechanical Characteristics of Preheated Composite Used for Cementation

Most studies compared flowable resin composite, preheated resin composite, and dual-cured self-adhesive resin cement, as they are the most commonly used luting agents, especially for thin preparations. Six studies showed preheated composite values to be similar to or surpass the other types of materials, with additional advantages such as closer interaction between the luting agent and the adhesive layer and better marginal adaptation and sealing. Thus, positive outcome research provides more reliability, with a higher specimen number and reliable results.

Castro-Ramírez et al. [28] evaluated the performance of flowable resin composite, preheated resin composite and dual-cured self-adhesive resin cement as dentin luting agents. The preheated resin composite demonstrated a significantly higher dentin microtensile bond strength than the dual-cured self-adhesive resin cement. No significant differences were observed between the flowable resin composite and either the preheated composite or the dual-cured self-adhesive cement. Use of the preheated resin composite at 70 °C yielded the highest dentin bond strength.

Contrary to this, another study showed no influence of the heating temperature on dentin adhesion. The flexural strength, shear bond strength, and interfacial tension of three types of preheated composite resins were investigated at temperatures of 25, 37, 54, and 68 °C. The tested luting surfaces included glass-ceramic and human dentin substrates. Preheated Tetric EvoCeram demonstrated a higher flexural strength compared to the control group at 25 °C, while Filtek Supreme XT exhibited greater flexural strength than Tetric EvoCeram. For shear bond strength to dentin, Filtek Supreme XT achieved higher values than the other materials. Although the preheating temperature did not influence the shear bond strength to dentin, it positively affected bonding to glass-ceramic, where Tetric EvoCeram showed significantly higher values than Venus and Filtek Supreme XT. Additionally, interfacial tension increased significantly with rising preheating temperatures [31].

Aiming to test both enamel and dentin bond characteristics, third molars were prepared to receive composite resin restorations with cavity depths of 2 mm and 4 mm. These restorations were luted using one resin cement and two composite resins. The composite resins were evaluated at room temperature and after preheating to 64 °C. For the 2 mm restorations, the composite resin Z250 XT—whether used at room temperature or preheated—showed significantly higher microtensile bond strength compared to the RelyX ARC resin cement. At this depth, Venus exhibited no significant difference compared to the resin cement; however, in the 4 mm restorations, only the preheated Venus demonstrated significantly higher bond strength than RelyX ARC. These findings lead to the conclusion that preheating the composite resin produces thinner luting interfaces and a closer interaction between the luting agent and the adhesive layer [30].

Different luting procedures and restoration thicknesses affect the flexural strength of CAD/CAM restorations. Lithium disilicate and resin composite restorations were bonded to dentin-analog disks using various materials: dual-curing resin cement (Panavia V5), light-curing resin cement (Panavia Veneer LC), preheated resin composite (Clearfil™ AP-X) with or without preheated restorations, and flowable composite (Clearfil Majesty™ Flow). The light-curing cement achieved the highest flexural strength, followed by dual-curing cement, preheated composite with preheated restoration, preheated composite alone, and flowable composite. For thicker lithium disilicate specimens, light-curing cement and preheated composite yielded similar results, while in thinner specimens, all luting methods performed comparably. In thin resin composite discs, light-curing cement provided superior strength, whereas thickness reduced the effect of luting type [32]. Light-cured composites have mechanical properties influenced by rate of conversion, and therefore one explanation for the outcome of this study may be inadequate photopolymerization through a thicker restoration.

While examining the effects of immediate versus delayed dentin sealing, one study [26] evaluated lithium disilicate overlays bonded to maxillary premolars using either preheated composite, dual-cure adhesive resin, or flowable composite. The subgroup that used preheated composite with immediate dentin sealing demonstrated the highest mean fracture resistance, whereas the subgroup with delayed dentin sealing and flowable composite showed the lowest mean value—a difference that was statistically significant, showing the superior outcome obtained from the use of preheated composites with higher mechanical properties than flowable ones.

When considering microleakage, no significant difference was observed between the preheated composite resin or self-adhesive resin cement in bonding indirect composite inlays to human premolars. However, self-adhesive cement produced higher microtensile bond strength, while preheated resin provided better marginal adaptation and sealing [33].

Three studies, two focused on bonding to enamel surfaces and one to dentin analog surfaces, contradicted these positive findings. They showed lower bond strength, increased film thickness, and a reduced degree of polymerization for the preheated composite.

One study [27] aimed to evaluate and compare the shear bond strength of light-cured resin cement, preheated composite resin, and dual-cured self-adhesive resin cement in bonding lithium disilicate disks to human premolar enamel. The results revealed, in a statistically significant manner, that the light-cured resin cement group exhibited the highest shear bond strength, followed by the dual-cured resin cement group, whereas the preheated composite resin group demonstrated the lowest values.

Investigating the effect of preheating resin-based materials and ultrasound application on the failure load of lithium disilicate glass-ceramic bonded to dentin analog specimens led to similar results. The analysis of failure load revealed no statistically significant differences among groups with respect to luting agent type, application method, or their interaction. The authors concluded that neither preheating resin-based materials nor ultrasound application influenced the failure load of lithium disilicate glass-ceramic; however, lower reliability was observed when the supra-nanofilled resin composite was preheated [29].

Another study [34] evaluated the polymerization quality and bond joint thickness of a dual-component adhesive and a heated composite resin when bonding ceramic onlays of varying thicknesses to human premolars, sectioned apically. The Vickers hardness of a dual-polymerizing cement was lower compared with the heated light-cured cement. Moreover, the thickness of the resin–ceramic restoration did not significantly affect the polymerization of a dual-polymerizing cement, but the polymerization of the preheated light-cured composite resin in the thickest was significantly lower. In addition, the mean film thickness of the dual-polymerizing groups was significantly lower than that of the heated light-polymerizing groups. A temperature reduction of 15 °C in the heated composite resin was also observed after 8 s.

These contradictory findings are mainly caused by different working protocols, as preheated composite luting is highly technique-sensitive. Light-cured composites are most indicated in thinner restorations, as a higher degree of polymerization can be achieved.

### 4.2. Other Characteristics of the Preheated Composite Used for Cementation

Marginal discrepancies in cementation, color stability and pulpal temperature changes are also main focus areas for the research of preheated composite.

Therefore, an in vitro study assessed the effect of different resin luting cements on the vertical marginal discrepancy of lithium disilicate pressed crowns and found that crowns cemented with preheated composite resin exhibited marginal gaps exceeding clinically acceptable limits [37]. These findings are consistent with evidence that the use of preheated composites as luting agents requires a higher level of clinical expertise to achieve an acceptable film thickness. In support of this, experiments involving human dentin and composite disks luted with two dual-cured cements and two light-cured preheated composites demonstrated that achieving clinically satisfactory results with preheated composites necessitates greater technical skill [39].

The objective of this study was to evaluate the color stability of ceramic veneers luted with resin cements and preheated composite resins (60 °C) over a 12-month period, and to determine the degree of conversion of the luting agents. Two types of resin cement—light-cured and dual-cured—and three types of composite resin—minifilled, microhybrid, and microfilled—were employed for cementing lithium–silicate glass-ceramic laminates onto bovine enamel substrates. No statistically significant differences were observed among the materials with respect to the degree of conversion. Preheating of the composite resins did not result in a higher degree of conversion and had no influence on the color stability of the cemented veneers. These findings indicate that not all composite resins are suitable for preheating and veneer cementation without potential alterations in their physical and mechanical properties. Consideration should be given to the composition, filler content, and photoinitiator system of each material. Additionally, factors such as ceramic thickness and marginal fit must be accounted for, as more flowable materials are preferable for thinner cement layers. The microfilled composite resin, both at room temperature and after preheating, exhibited clinically relevant color changes after 12 months of storage. Overall, preheating did not adversely affect the color stability of the composite resins evaluated [35].

Different temperatures may provide different outcomes, as shown in a 2023 study that tested maxillary premolars bonded to either lithium disilicate or zirconia using a dual-cure resin cement at two different temperatures: 25 °C and 54 °C. Following cementation, the specimens were aged and immersed in coffee to simulate staining conditions. Restorations cemented at 54 °C exhibited a significantly lower color difference compared with those cemented at 25 °C, indicating that elevated cementation temperature may enhance the color stability of both lithium disilicate and zirconia restorations [38].

Preheated composite resins cool very quickly after being removed from the heating device. One study found that a resin preheated to 68 °C lost 45–61% of its temperature within 15 s, 84% within 30 s and 96% within 60 s, suggesting a very short window (10–15 s) for the ideal seating of indirect restorations [8]. Composites heated to 60 °C regained viscosity rapidly, and 5 min after removal from the heater they cooled down to room temperature. Therefore, when using preheated composites for luting, the restoration must be seated and adapted immediately (ideally within 30–60 s) to take advantage of the lowered viscosity [8].

The luting of thin ceramic indirect restorations may result in an increase in pulpal temperature, potentially leading to pulpal injury due to the heat generated by the light-curing unit and the exothermic polymerization reaction of the luting agent. Two studies focused on this aspect were identified. One study used light-cured, dual-cured adhesive cements and preheated restorative resin-based composites for the luting of lithium disilicate ceramic blocks of varying thicknesses. The remaining dentin thickness from the pulpal chamber wall measured 2.5, 2.0, 1.5, and 1.0 mm, respectively. Temperature changes were primarily affected by dentin thickness, followed by the type of luting agent and ceramic thickness. Irrespective of ceramic thickness, adhesive inlay cementation was found to significantly elevate pulpal temperature, particularly when the residual dentin thickness was less than 2 mm [36]. A second study investigated pulpal temperature changes in bovine teeth with veneer-type preparations during cementation using preheated composite or light-cured resin cement reported that specimens cemented with preheated composite and those with the greatest preparation depth exhibited the highest mean intrapulpal temperatures [14].

### 4.3. Limitations of This Study and Further Research

This scoping review presents itself with a series of limitations. Firstly, the heterogeneity of studies, with their variations in design, sample size, and methodology, rendered comparisons difficult. Secondly, the evidence included in our review was primarily derived from in vitro studies, and therefore may not fully replicate the clinical environment, including intraoral temperature fluctuations, occlusal loading, and long-term aging. Thirdly, this review is not a meta-analysis and does not provide a statistical synthesis of the findings, limiting the strength of the conclusions. Findings from certain studies, using bovine teeth or dentin analog materials, may not apply to all clinical situations.

Additionally, data on pulpal safety, long-term color stability, and clinical performance in high-stress areas remain limited. Future research should focus on standardized in vivo studies to evaluate the mechanical, optical, and biological outcomes of preheated composites under clinically relevant conditions. Investigations into optimal preheating temperatures, material-specific protocols, and techniques to minimize pulpal temperature rise would further support safe and effective clinical application.

### 4.4. Strengths of This Review

This scoping review possesses several noteworthy strengths that enhance the quality and relevance of its findings. First, the review adopts a clearly defined and focused scope by examining only studies performed on dentin and enamel substrates. This approach avoids the variability introduced by previous reviews that included non-dental surfaces such as plastic or composite blocks, thereby increasing the clinical relevance of the conclusions. Additionally, the review addresses a significant gap in the existing literature by evaluating mechanical, optical, and biological outcomes collectively. Previous work has tended to focus on only one of these domains, whereas this review provides a more comprehensive understanding of the potential benefits and limitations of using preheated composite as a luting material.

This review reflects contemporary materials and techniques published in the last 10 years. By requiring that included studies compare preheated composites with at least one other cement type, this review ensures that all selected articles contribute meaningful comparative data rather than merely descriptive observations.

Furthermore, the emphasis on dentin, enamel, and their accepted analogs reduces substrate variability and contributes to more reliable and interpretable outcomes. The inclusion of multiple outcome measures offers a multidimensional perspective on the clinical viability of preheated composite resins. Collectively, these strengths position the review as a timely and clinically relevant contribution to the field of adhesive dentistry, helping guide future research and informing evidence-based decision-making in restorative practice.

## 5. Conclusions

This scoping review aimed to map the existing evidence on the use of preheated composite resins as luting agents for indirect restorations and to clarify their mechanical, optical, and biological implications. The findings indicate that preheated composites can offer meaningful advantages, including improved mechanical performance, enhanced interfacial adaptation, and acceptable optical behavior, particularly when used on dentin and glass-ceramic substrates.

From a clinical perspective, preheated composites may be considered a viable alternative to conventional resin cements in selected cases, provided that meticulous technique is employed. Clinicians should carefully control restoration thickness, ensure adequate polymerization, and consider remaining dentin thickness to minimize the risk of excessive intrapulpal temperature rise. Material selection, including composite formulation and viscosity, remains critical to achieving predictable outcomes.

Despite these advantages, important gaps in the literature remain. Evidence is inconsistent regarding bonding performance on enamel, polymerization efficiency in thicker restorations, and long-term clinical outcomes. There is also limited standardization in preheating protocols, outcome measures, and testing methodologies, which restricts direct comparison across studies.

Future research should prioritize well-designed in vitro and clinical studies with standardized preheating temperatures, restoration thicknesses, and outcome measures. Long-term randomized clinical trials are particularly needed to evaluate durability, biological safety, and esthetic stability under functional conditions. Addressing these gaps will help establish evidence-based guidelines for the safe and effective clinical use of preheated composite resins as luting agents.

## Figures and Tables

**Table 1:** Search concepts.

Concept	Keywords and MeSH Terms
Preheated AND composite AND luting	“preheat*” [Tw] AND “composit*” [Tw] AND “lut*” [Tw]
Preheated AND luting	“preheat *” [tw] AND “Lut*”
Preheated AND composite AND cementation	“preheat*” [tw] AND “composit*” [Tw] AND “cement*” [tw]
Preheated AND cementation	“preheat*” [Tw] AND “cement*” [Tw]

**Table 2:** Search combinations per database.

Database	Search Terms and Combinations
PubMed Embase Scopus	“preheat*” [Tw] AND (“lut*” [Tw] AND “composit” [Tw]) OR (“cement” [tw] AND “composit” [Tw]) “preheat*” [tw] AND “cement” [tw] OR “lut*” [Tw]

**Table 3:** Inclusion and exclusion criteria.

Category	Inclusion Criteria	Exclusion Criteria
Study Focus/Groups	Articles comparing preheated composite with other cement types	Studies without at least two groups or without a preheated composite comparison
Study Design	In vitro studies	Clinical studies and reviews
Substrate Type	Performed on dentin or enamel substrates (human, bovine, or analog)	Studies evaluating bonding to composite, plastic, or other non-dental surfaces
Language	Published in English	Articles published in languages other than English
Publication Date	Published between 2015 and 2025	Articles published more than 10 years ago
Other	—	Studies lacking comparative analysis

**Table 4:** Articles on mechanical behavior.

	Author, Year	Luting Surfaces	Luting Agents	Results
1	Abdulsattar and Kadhim [26]	Dentine/lithium disilicate	Delay dentin sealing (DDS)/immediate dentin sealing (IDS) + preheated composite or dual cure or flowable composite	Preheated composite had better fracture resistance, followed by resin cement and flowable composite. Fracture resistance for immediate dentin sealing and preheated composite—highest mean value.
2	Akyle and Achour [27]	Dentine and enamel/lithium disilicate	Light-cured resin cement, preheated composite resin, dual-cured self-adhesive resin cement	Light-cured resin cement—higher SBS compared to preheated resin composite and dual-cured resin cement with a self-etch system. Preheating composite resins may increase mechanical properties and improve luting ceramics.
3	Castro-Ramirez, Ladera-Castañeda, Cachay-Criado, Alvino-Vales, López-Gurreonero, Cervantes-Ganoza and Cayo-Rojas [28]	Dentin/resin blocks	Flowable resin composite, preheated resin composite, and dual self-adhesive resin cement	Preheated resin composite had significantly higher microtensile bond strength compared to dual self-adhesive cement; flowable resin composite had no significant difference from dual self-adhesive cement or preheated resin composite. Microtensile bond strength in dentin—significantly higher with preheated resin composite at 70 °C compared to dual self-adhesive, but similar to flowable resin composite.
4	Figueiredo, Spazzin, Bacchi and Alessandretti [29]	Dentin analog/lithium disilicate	Light-cured luting agent, flowable resin composite, supra-nano filled resin composite + preheating or ultrasound	No significant difference among groups considering the type of luting agent, application method and interaction. Preheating and ultrasound application—no effect on failure load of lithium disilicate glass-ceramic, but lower reliability for supra-nano filled resin composite.
5	Goulart, Borges Veleda, Damin, Bovi Ambrosano, Coelho de Souza and Erhardt [30]	Dentin and enamel/composite	One resin cement, two composite resins—room temperature and preheated to 64 °C	For 2 mm restorations, composite resin, preheated or not, had significantly higher microtensile bond strength than the resin cement, with the other composite equal. 4 mm restorations, preheated composite—significantly higher microtensile bond strength than dual cure cement. Preheating composite resin had thinner luting interfaces.
6	Kramer, Edelhoff and Stawarczyk [31]	Glass-ceramic/dentin	Three composite materials, different temperatures.	Higher temperature—positive on adhesion; on one group of SBS tests, higher values for preheated composites. Improved adaptation after preheating, less microleakage as lower viscosity—better surface penetration; fracture patterns on dentin were mainly adhesive.
7	Tribst, Etoeharnowo, Tadros, Feilzer, Werner, Kleverlaan and Dal Piva [32]	Lithium disilicate or resin composite/dentin analog	Dual-curing resin cement/light-curing resin cement/preheated resin composite/high-filled flowable composite with or without preheated restoration	Luting procedures impact the flexural strength of CAD/CAM lithium disilicate and resin composite. Preheated resin composite can replace dual-curing cement; light-curing cement has superior performance for specific thicknesses, but is thicker—less sensitive to luting variations.
8	Urcuyo [33]	Resin composite/dentin	Preheated composite/self-adhesive resin cement	No significant difference in the degree of microfiltration; microtensile bond strength—greater for resin cement; better adjustment and sealing for preheated composite.
9	Secundar [34]	Lithium disilicate/enamel, dentin	Dual-component adhesive/preheated composite resin	Vickers hardness of dual resin cement—lower compared to preheated light-cure cement; polymerization of the preheated light-cure composite resin for high thickness onlay—significantly lower; mean film thickness of the dual-polymerizing—significantly lower.

**Table 5:** Articles focused on non-mechanical outcomes of preheated composite.

	Author, Year	Luting Surfaces	Luting Agents	Results
1	Gugelmin, Miguel, Baratto Filho, Cunha, Correr and Gonzaga [35]	Bovine enamel/lithium-silicate	Two resin cements, light-cured and dual-cured/three composite resins, minifilled, micro-hybrid, and microfilled	Different luting agents influenced the final color of the restorations; heating had no effect on the degree of conversion. Light-cured and dual-cured resin cements had similar color stability; microfilled composite resin at room temperature and preheated showed clinically relevant color change after 1 year. Heating had no effect on the color stability.
2	Kincses, Jordáki, Szebeni, Kunsági-Máté, Szalma and Lempel [36]	Dentin/lithium disilicate ceramic	Light-cured/dual-cured adhesive cements/preheated restorative resin-based composite	Pulpal temperature values influenced by the remaining dentin thickness, by the applied resin-based adhesive luting materials, and least by ceramic thickness. Preheated composite raised pulpal temperature to the highest value, not significantly different.
3	Mounajjed, Salinas, Ingr and Azar [37]	Lithium disilicate/dentin, enamel	Resin cement, flowable resin composite, high viscosity composite resin	The least amount of marginal increase after cementation—flowable composite; the highest marginal increase—preheated composite resin. Also exceeded clinically acceptable range of marginal discrepancy.
4	Sakrana, Laith, Elsherbini, Elerian, Özcan and Al-Zordk [38]	Lithium disilicate/zirconia/dentin/enamel	Resin cement G-CEM LinkForce/Panavia SA Cement Plus Automix—preheating temperature 25 °C or 54 °C + thermocycling Immersed in coffee	Cement at a temperature up to 54 °C enhances the color stability of lithium disilicate and zirconia restorations.
5	Teyagirwa, Aquin, Kharouf, Roman, Senger, Reitzer and Etienne [39]	Dentin/composite disks	Two preheated composites, two resin cements	Preheated composites require a better level of expertise for a clinically acceptable film thickness.
6	Hatner [14]	Lithium disilicate/enamel, dentin bovine	Preheated composite resin, photopolymerizable resin cement	Preheated composite resin + deepest preparation—highest mean intrapulpal temperature, up to 5.70 ± 2.14 °C.

## Data Availability

No new data was created or analyzed in this study. Data sharing is not applicable to this article.

## Supplementary Materials

The following supporting information can be downloaded at: https://www.mdpi.com/article/10.3390/dj14010069/s1, File S1: PRISMA-ScR (Preferred Reporting Items for Systematic Reviews and Meta-Analyses Extension for Scoping Reviews) Checklist.
