# Supplementary material for: Preheated Composite for Prosthetic Cementation to Enamel and Dentin: A Scoping Review
Source: Dent J (Basel). 2026 Jan 21;14(1):69. doi: 10.3390/dj14010069 (PMC12839924; doi:10.3390/dj14010069)
Supplement: Supplementary file 1 [file dentistry-14-00069-s001.zip › dentistry-3962987-supplementary.pdf]

PRISMA-ScR (Preferred Reporting Items for Systematic Reviews and Meta-Analyses Extension for Scoping Reviews) Checklist

This checklist is adapted from Tricco et al. (2018) to help ensure transparency and completeness of our scoping review.

Section	Item	Checklist Item	Reported on Page #
TITLE	1	Identify the report as a scoping review.	1/ line 2
ABSTRACT	2	Provide a structured summary including background, objectives, eligibility criteria, sources of evidence, charting methods, results, and conclusions.	2/ lines 19-40
INTRODUCTION	3	Rationale: Describe the rationale for the review in the context of what is already known. Explain why a scoping review approach was chosen.	3/ lines 99-103
INTRODUCTION	4	Objectives: Provide an explicit statement of the questions and objectives being addressed, with reference to key elements (population, concept, context).	3/ lines 96-99, 103-108
METHODS	5	Protocol and registration: Indicate if a review protocol exists, where it can be accessed (e.g., OSF, PROSPERO*), and registration number if applicable. (*Note: PROSPERO generally does not accept scoping reviews.)	Not applicable
METHODS	6	Eligibility criteria: Specify characteristics of sources of evidence (e.g., years considered, language, publication status) and rationale.	4/ subsection 2.2

METHODS	7	Information sources: Describe all information sources (e.g., databases, websites, grey literature) and date last searched.	4/ subsection 2.1
METHODS	8	Search: Present the full electronic search strategy for at least one database, including any limits used.	4/ Table 1, 2
METHODS	9	Selection of sources of evidence: State the process for selecting sources (screening, eligibility, inclusion).	5/ subsection 2.1, 2.3
METHODS	10	Data charting process: Describe the methods of data extraction ("charting"), including who did it and how discrepancies were resolved.	5/ lines 150-160
METHODS	11	Data items: List and define all variables for which data were sought (e.g., study design, population, concepts, context, outcomes).	4/ subsection 2.2 – criteria 3 – inclusion and 4 - exclusion
METHODS	12	Critical appraisal of individual sources (if done): If you assessed source quality, describe rationale and methods used.	5/ lines 157-160
METHODS	13	Synthesis of results: Describe the methods used to summarize or present the charted data.	5/ subsection 2.3
RESULTS	14	Selection of sources of evidence: Provide numbers of sources screened, assessed for eligibility, and included, with reasons for exclusions — ideally with a flow diagram.	5/ subsection 3.1, Figure 1 – PRISMA flow diagram
RESULTS	15	Characteristics of sources: Present characteristics for each included	8-10/ Tables 3, 4

		source (e.g., study design, setting, participants, concept).	
RESULTS	16	Critical appraisal within sources (if done): Present results of critical appraisal if performed.	Not applicable
RESULTS	17	Results of individual sources of evidence: Present relevant data from each included source that relate to the review questions.	6/ lines 182-188
RESULTS	18	Synthesis of results: Summarize the main findings in relation to the review objectives, such as themes or knowledge gaps identified.	6/ lines 178-181
DISCUSSION	19	Summary of evidence: Summarize the main results (including concepts, themes, and types of evidence available), linking to the objectives and questions.	7/ lines 212-219
DISCUSSION	20	Limitations: Discuss limitations of the scoping review process.	13/ subsection 4.6
DISCUSSION	21	Conclusions: Provide general interpretation of the results with implications for future research, practice, and/or policy.	14/ section 5
FUNDING	22	Funding: Describe sources of funding for the included evidence and for the scoping review itself, and the role of funders.	14/ lines 410-411
